# Spread of SARS-CoV-2 Infection in Adult Populations in Cameroon: A Repeated Cross-Sectional Study Among Blood Donors in the Cities of Yaoundé and Douala

**DOI:** 10.1007/s44197-023-00102-7

**Published:** 2023-05-02

**Authors:** Arsène Brunelle Sandie, Françoise Ngo Sack, Christiane Ingrid Medi Sike, Joseph Mendimi Nkodo, Hortense Ngegni, Haverie Ghislaine Ateba Mimfoumou, Sarah Audrey Lobe, Diane Choualeu Noumbissi, Fabrice Tchuensou Mfoubi, Paul Alain Tagnouokam Ngoupo, Lawrence Ayong, Richard Njouom, Mathurin Cyrille Tejiokem

**Affiliations:** 1 Service d’épidémiologie Et de Santé Publique, Centre Pasteur du Cameroun, 451, Street 2005, Yaounde 2, P.O. Box: 1274, Yaoundé, Cameroon; 2 African Population and Health Research Center, Dakar, Senegal; 3 Hôpital Central de Yaoundé, Yaoundé, Cameroon; 4 Hôpital Laquintinie de Douala, Douala, Cameroon; 5 Hôpital Jamot de Yaoundé, Yaoundé, Cameroon; 6 Service de Virologie, Centre Pasteur du Cameroun, Yaoundé, Cameroon; 7 Service de Paludisme, Centre Pasteur du Cameroun, Yaoundé, Cameroon

**Keywords:** COVID-19, Cameroon, Adult population, Repeated cross-sectional, Seroprevalence, Wide spreading, Anti-SARS-CoV-2 antibody

## Abstract

Over a period of about 9 months, we conducted three serosurveys in the two major cities of Cameroon to determine the prevalence of SARS-COV-2 antibodies and to identify factors associated with seropositivity in each survey. We conducted three independent cross-sectional serosurveys of adult blood donors at the Central Hospital in Yaoundé (CHY), the Jamot Hospital in Yaoundé (JHY) and at the Laquintinie Hospital in Douala (LHD) who consented in writing to participate. Before blood sampling, a short questionnaire was administered to participants to collect their sociodemographic and clinical characteristics. We included a total of 743, 1202, and 1501 participants in the first (January 25–February 15, 2021), second (May 03–28, 2021), and third (November 29–December 31, 2021) surveys, respectively. The adjusted seroprevalence increased from 66.3% (95% CrI 61.1–71.3) in the first survey to 87.2% (95% CrI 84.0–90.0) in the second survey, and 98.4% (95% CrI 96.8–99.7) in the third survey. In the first survey, study site, participant occupation, and comorbid conditions were associated with SARS-CoV-2 seropositivity, whereas only study site remained associated in the second survey. None of the factors studied was significantly associated with seropositivity in the third survey. Together, the data suggest a rapid initial spread of SARS-CoV-2 in the study population, independent of the sociodemographic parameters assessed.

## Introduction

Since December 2019, the coronavirus disease 2019 (COVID-19) has increasingly spread to all countries worldwide. It began in Wuhan in China and quickly spread around the world, to a level that multiple waves of the outbreak have been observed to date. According to the reported data, four waves of the outbreak have been observed worldwide and more than 578 million individuals have been infected with more than 6 million deaths [1]. Reported figures on the COVID-19 outbreak are generally based on routine laboratory data. Such numbers are generally skewed and underestimated [2, 3]. This would be particularly the case for the African region which, based on the reported data, is the least affected region in the world, with a cumulative total of about 9 million cases, representing only 2% of total cases. Given the low testing capacity and the largely young and asymptomatic population, these numbers do not represent the actual situation of the outbreak in this region [4–6]. Seroprevalence studies have been carried out in many settings, including African countries, to better estimate the actual situation of the outbreak.

One finds that in most African countries, seroprevalence studies have been conducted mostly during the first wave of the outbreak. A meta-analysis in [7] provides an overall seroprevalence estimate of 22% for African countries during the first wave of the outbreak. Based on reported data, Cameroon has recorded a cumulative number of about 120,000 cases of COVID-19 to date [1]. To date, no nationwide serosurvey has been carried out to assess the true extent of the outbreak. However, some seroprevalence studies in Yaoundé have suggested a widespread of the virus in the population. A study by Nwosu et al. [8] conducted in Yaoundé during the first wave estimated seroprevalence at 29.2%. Another seroprevalence study conducted by Ndongo et al. [9] in Yaoundé during the second wave, specifically during the months of April–May 2021, estimated seroprevalence at 51.3%. Few studies in this setting have been carried out to evaluate the dynamics of the spread of the virus. However, repeated seroprevalence surveys carried out in some African settings [9–12] during the first and second waves have indicated both high seroprevalence (reaching 70% in certain cases) and rapid increases in seroprevalence estimates. To date, no known repeated seroprevalence studies in African settings have been carried out to evaluate the extent of the virus spread alongside the drivers or associated factors of spread over time. The overall objective of this study was to assess the extent of anti-SARS-CoV-2 antibody seroprevalence over a period of about 9 months, with three periodic repeated seroprevalence surveys in three COVID-19 referral diagnostic centers located in Yaoundé and Douala, two major cities in Cameroon.

## Methods

### Study Design

**Fig. 1:**
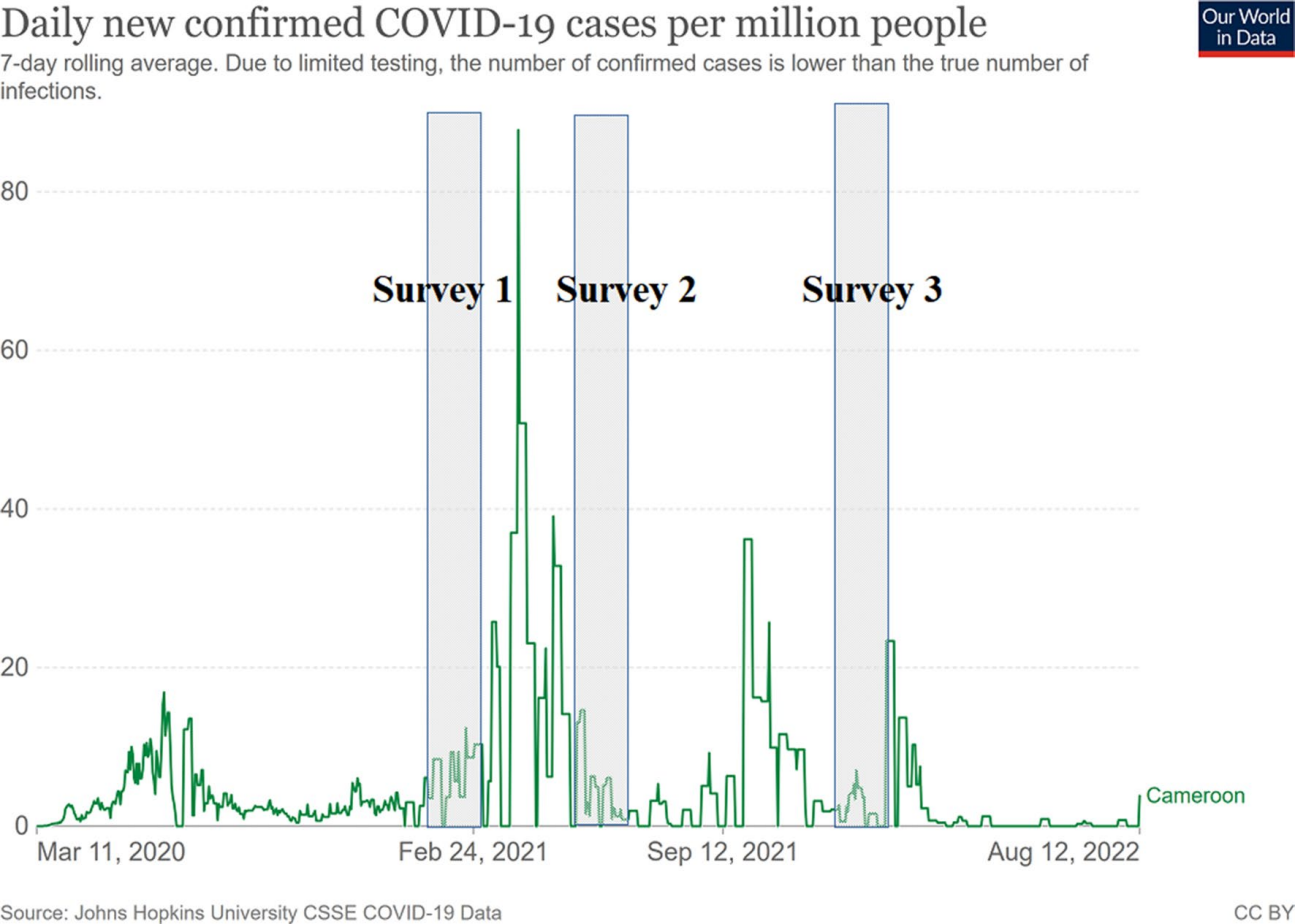
Trend of COVID-19 cases in Cameroon (Sourced from John Hopkins University CSSE COVID-19 Data) and the three serosurveys period of our study

This was a repeated independent cross-sectional seroprevalence survey conducted at three different periods in 2021: January 25–February 15 (survey 1), May 03–28 (survey 2), and November 29–December 31 (survey 3). Figure 1 shows the trend in the number of reported COVID-19 cases in Cameroon and the periods during which our serosurveys were carried out. Survey 1 was conducted at the early stage of the second wave, survey 2 at the end of the second wave, and survey 3 at the end of the third wave. Three sites in the two major cities (Yaoundé and Douala) of Cameroon were selected for the serosurveys; these were the Blood Donors Services at the Central Hospital of Yaoundé (CHY) and the Jamot Hospital of Yaoundé (HJY) for Yaoundé and the Laquintinie Hospital in Douala (LHD) for Douala, respectively. It was a consecutive and observational sample, where all healthy individuals aged from 18 to 55 years at all sites were included in the study after consent provision.

### Blood Sampling and Data Collection

For each biological sample collected, the time of collection, transport conditions, and time of arrival at the study laboratory were recorded. Blood collection was designed to ensure that specimens reached the Virology laboratory of Centre Pasteur du Cameroun as soon as possible after collection. At this point, the sample was centrifuged and sera collected and stored at − 80 °C. When the specimen was not likely to reach the laboratory within 72 h, the serum was separated from the whole blood before being stored at − 20 °C, and shipped on dry ice. Repeated freezing and thawing of specimens were generally avoided. Before blood collection, participants were interviewed on their sociodemographic variables (sex, gender, marital status, and type of work) and clinical history (comorbidities, being COVID-19 before, being vaccinated) using a structured quantitative questionnaire.

### COVID-19 Serological Assays (ELISA)

Serological analysis was performed by batch at the end of the study using the Wantai SARS-CoV-2 IgG ELISA Kit (quantitative) for detection of total anti-SARS-CoV-2 antibodies.

The Wantai ELISA was performed following the manufacturer's protocol. Briefly, after labeling three wells as negative calibrators, two wells as positive calibrators, and one well as blank, 50 µl of positive and negative calibrators and 100 µl of specimen were added to their respective wells, except for the blank. Then the plate was covered with a lid and incubated at 37 °C for 30 min. At the end of the incubation, the plate cover was removed and wells were washed five times with reduced wash buffer. After the wash cycle, the plate was inverted onto blotting paper and tapped to remove remaining wash solution, then 100 µl of HRP conjugated antibodies were added to each well except the blank. The plate was then covered and incubated at 37 °C for 30 min. After five additional washes, 50 µl of chromogenic solution A and 50 µl of chromogenic solution B were added to each well, including the blank. The plate was then covered and incubated at 37 °C for 15 min in the dark. At the end of the incubation, 50 µl of stop solution was added and the absorbance was read at 450 nm. The validation of the Wantaï SARS-CoV-2 Ab ELISA [13] indicated sensitivity and a specificity of 96.7% (95% CI 83.3–99.4) and 97.5% (95% CI 91.3–99.3), respectively. The interpretation of the results obtained was based on the calculation of the ratio between the value of the optical density of the sample (DOs) and that of the cut-off (CO) as proposed by the test manufacturer (ratio = DOs/CO). The test was considered negative if the ratio was ≤ 1.1.

### Statistical Analysis

Having obtained observational data, not derived from a representative sample survey, we used age and sex to construct post-stratified weights using census population projections. First, crude seroprevalences were estimated by relating the total number of seropositive samples to the total number of samples tested. Second, age- and sex-standardized seroprevalences and 95% confidence interval (CI) were estimated using post-stratified weights. Furthermore, the estimated age–sex-standardized seroprevalence was adjusted to account for the test validity [14, 15]. However, when the apparent prevalence is greater than the sensitivity of the test (as this would be the case for some stratifiers in survey 3), the classical prevalence adjustment would be invalid as discussed by Stevenson et al. [16] In such cases, the Bayesian approach is recommended for seroprevalence estimation taking into account the test validity. Thus, the adjusted seroprevalences with their 95% credible intervals (CrI) were estimated as described in Speybroeck et al. [17].

The average monthly percent change (AMPC) [18, 19] in adjusted seroprevalence and its confidence intervals were estimated to evaluate the spread of the virus over time. The time points: 5th February, 16th May, and 14th November 2021, median periods of January 25–February 15 (survey 1 period), May 03–28 (survey 2 period), and November 29–December 31 (survey 3 period), respectively, were considered the time points surveys. These time points were considered as the joint point in the AMPC estimations, which corresponds to a period of 3.29 months from surveys 1 to 2, and 5.95 months from surveys 2 to 3.

For each survey, the Chi-square test for independence was used to test the independence of seropositivity with sociodemographic and clinical variables, respectively. A weighted logistic regression model was finally used to identify factors associated with seropositivity after investigating potential confounders. All statistical analyses were done with R version 4.0.4., packages [16, 20, 21] “survey”, “epitabulate” and “epiR” were particularly used in the analysis.

## Results

### Population Characteristics

Table 1 summarizes the distribution of the population sampled according to the site and sociodemographic variables. A total of 3446 individuals were sampled on the 3 sites and for the 3 surveys (743, 1202, and 1501 for surveys 1, 2, and 3, respectively). Overall, CHY, JHY, and LHD sampled 1650, 873, and 923 individuals, respectively, and the distribution of participants according to site in the three surveys was significantly different (*p* value < 0.001). The sociodemographic characteristics of the sampled population were similarly distributed in the three surveys. The participants were predominantly male (90.3%), generally young aged 18–34 years (77.8%), and mostly singles (83.7%). Most of them were either students (34.3%) or technicians/agents (30.5%), a few held senior positions (12.1%), and the remaining 23.1% were classified in various professional categories such as musician, footballer, domestic worker, etc.

**Table 1:** Distribution of participants according to the study site and sociodemographic characteristics, Blood Donors Services, Cameroon, 2021

	Survey 1	Survey 2	Survey 3	Pooled surveys	*p* value
Total	*N* = 743	*N* = 1202	*N* = 1501	*N* = 3446	
Study site	% (*n*)	% (*n*)	% (*n*)	% (*n*)	< 0.001
CHY	54.5 (405)	43.2 (519)	48.4 (726)	47.9 (1650)	
JHY	22.5 (167)	28.4 (342)	24.2 (364)	25.3 (873)	
HLD	23.0 (171)	28.4 (341)	27.4 (411)	26.8 (923)	
Gender					0.9
Female	9.6 (71)	9.4 (113)	9.9 (149)	9.7 (333)	
Male	90.4 (672)	90.6 (1089)	90.1 (1352)	90.3 (3113)	
Grouped age					0.4
18–34	76.6 (569)	77.4 (930)	78.8 (1183)	77.8 (2682)	
35–55	23.4 (174)	22.6 (272)	21.2 (318)	22.2 (764)	
Marital status					0.7
Married/cohabiting	15.9 (118)	16.5 (198)	15.4 (231)	15.9 (547)	
Single	83.7 (622)	82.9 (997)	84.2 (1264)	83.7 (2883)	
Divorced/widow	0.4 (3)	0.6 (7)	0.4 (6)	0.5 (16)	
Occupation					0.10
Student	30.3 (225)	35.4 (425)	35.4 (532)	34.3 (1182)	
Technician/agent	33.0 (245)	30.5 (367)	29.2 (438)	30.5 (1050)	
Senior	14.1 (105)	11.6 (139)	11.5 (173)	12.1 (417)	
Other	22.6 (168)	22.5 (271)	23.9 (358)	23.1 (797)	

### Clinical Background

Table 2 summarizes the distribution of the population sampled according to participants’ clinical characteristics. In survey 1, the participants were more symptomatic (34.2%, 18.1%, and 22.8% for surveys 1, 2, and 3 respectively, *p* value < 0.001) and with more comorbidities than the participants in the other surveys (2.3%, 0.6%, and 0.8% for surveys 1, 2, and 3, respectively, *p* value < 0.001). However, in the pooled population of participants, the comorbidities proportion was very low and was estimated at 1%, while the pooled proportion of symptomatic was estimated at 23.6%. Across the three surveys, the distribution of participants who had a history of previous contact with confirmed COVID-19 cases was similar, with a pooled proportion of 1.2%. Regarding vaccination against COVID-19, in surveys 1 and 2, there were no vaccinated participants. In fact, COVID-19 vaccination in Cameroon was launched on April 12, 2021, only a few weeks prior to the start of survey 2, and at the start of vaccination, very few people were being vaccinated. In survey 3, 25 (1.7%) individuals were vaccinated including 11 (44%) with Johnson & Johnson, 8 (32%) with AstraZeneca, 5 (20%) with Sinopharm, and only 1 (4%) with Pfizer. Few participants had been previously tested for COVID-19 by PCR, a total of 377 (10.9%) in the pooled survey, in surveys 1 and 2, 12.8% of participants had been tested previously, this percentage was 8.5% in survey 3. Of the 377 who had been previously tested, only 16 (4.2%) were positive.

**Table 2:** Distribution of participants according to their clinical history, Blood Donors Services, Cameroon, 2021

	Survey 1	Survey 2	Survey 3	Pooled surveys	*p* value
Total	*N* = 743	*N* = 1202	*N* = 1501	*N* = 3446	
Comorbidities	% (*n*)	% (*n*)	% (*n*)	% (*n*)	< 0.001
None	97.7 (726)	99.4 (1195)	99.2 (1489)	99.0 (3410)	
One or more	2.3 (17)	0.6 (7)	0.8 (12)	1.0 (36)	
Symptoms					< 0.001
None	65.8 (489)	81.9 (984)	77.2 (1159)	76.4 (2632)	
One or more	34.2 (254)	18.1 (218)	22.8 (342)	23.6 (814)	
Previous contact with a COVID-19 case					0.14
Yes	0.8 (6)	1.8 (21)	1.1 (16)	1.3 (43)	
No/Do not know	99.2 (737)	98.3 (1181)	98.9 (1485)	98.8 (3403)	
COVID-19 vaccination	*	*	1.7 (25)	0.7 (25)	< 0.001
Type of vaccination					0.9
AstraZeneca	*	*	32.0 (8)	32.0 (8)	
Johnson&Johnson	*	*	44.0 (11)	44.0 (11)	
PFIZER	*	*	4.0 (1)	4.0 (1)	
SINOPHARM	*		20.0 (5)	20.0 (5)	
Previously PCR COVID-19 tested					< 0.001
Yes	12.8 (95)	12.8 (154)	8.5 (128)	10.9 (377)	
No	87.2 (648)	87.2 (1048)	91.5 (1373)	89.1 (3069)	
Number of previous PCR COVID-19 test realized					0.009
None	87.2 (648)	87.2 (1048)	91.5 (1373)	89.1 (3069)	
Once	7.7 (57)	8.2 (99)	5.6 (84)	7.0 (240)	
Twice	3.2 (24)	2.7 (33)	1.9 (29)	2.5 (86)	
Thrice +	1.9 (14)	1.8 (22)	1.0 (15)	1.5 (51)	
Outcome of previous test					0.14
Negative	98.9 (94)	95.4 (144)	93.9 (123)	95.8 (361)	
Positive	1.1 (1)	4.6 (7)	6.1 (8)	4.2 (16)	

*There was no vaccination at surveys 1 and 2

### Seroprevalence of Anti-SARS-CoV-2 Antibody

**Fig. 2:**
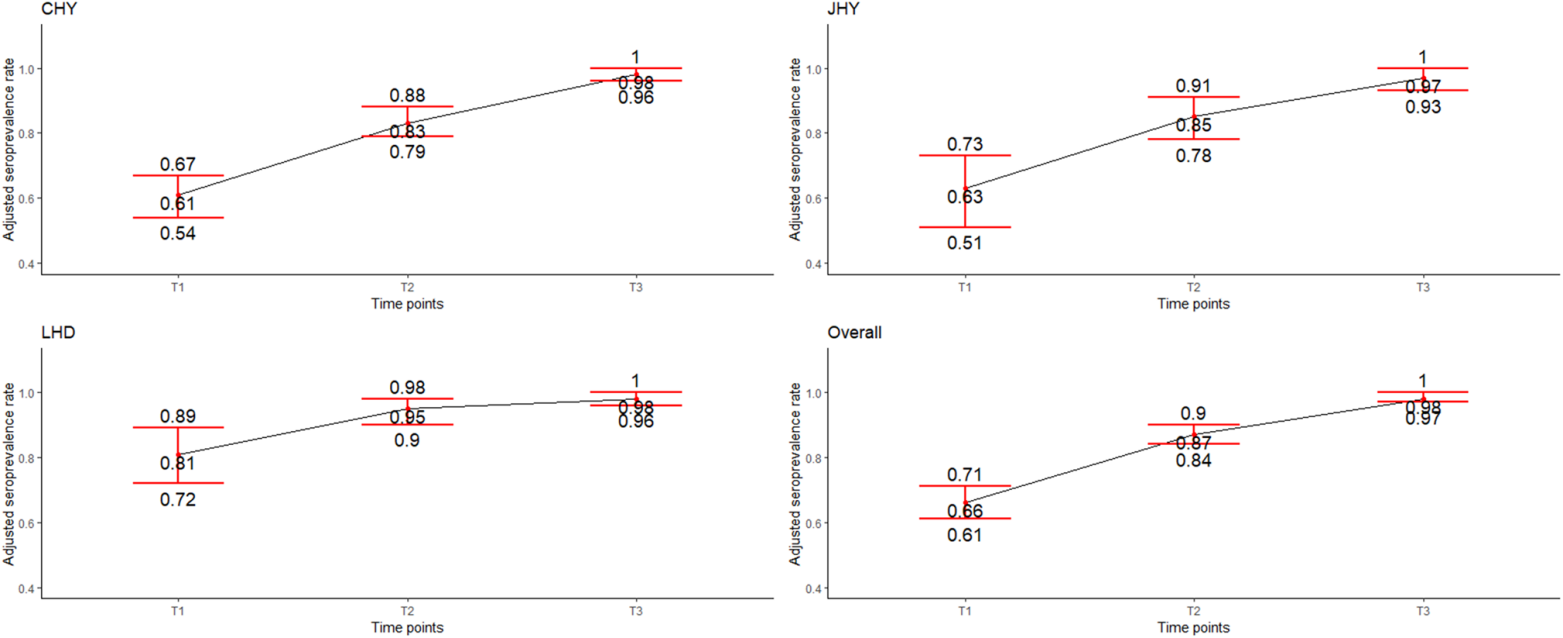
Anti-SARS-CoV-2 antibody seroprevalence estimated over time by study site and in the overall sample, Blood Donors Services, Cameroon, 2021

Figure 2 shows the trend in adjusted seroprevalence by site and in the overall sample, while Table 3 provides more detailed statistics. Overall, adjusted seroprevalence increased from 66.3% (95% CrI 61.1–71.3) in the first survey to 87.2% (95% CrI 84.0–90.0) in the second survey, and 98.4% (95% CrI 96.8–99.7) in the third survey. In survey 1, adjusted seroprevalence was not independent of the study site, participant profession, and comorbidity condition. In survey 2, only study site was associated with seroprevalence, while in survey 3, adjusted seroprevalence was independent of all covariates.

**Table 3:** Adjusted seroprevalence estimates according to site, sociodemographic and clinical variables, Blood Donors Services, Cameroon, 2021

	Survey 1				Survey 2				Survey 3			
	Positive tests	Age–sex-standardized seroprevalence (95% CI)	Adjusted seroprevalence (95% CrI)	*p* value	Positive tests	Age–sex-standardized seroprevalence (95% CI)	Adjusted seroprevalence (95% CrI)	*p* value	Positive tests	Age–sex-standardized seroprevalence (95% CI)	Adjusted seroprevalence (95% CI)	*p* value
Overall	475/742	64.0% (60.3–69.7)	66.3% (61.1–71.3)		1004/1201	84.7% (82.2–87.3)	87.2% (84.0–90.0)		1425/1500	95.3% (94.0–96.7)	98.4% (96.8–99.7)	
Study site				0.001				< 0.001				0.6
CHY	251/404	59.5% (52.8–65.9)	60.6% (53.6–67.5)		417/519	81.2% (76.3–85.3)	83.5% (78.8–87.9)		648/725	95.4% (93.5–96.7)	98.2% (96.0–99.8)	
JHY	97/167	62.2% (51.9–71.5)	62.7% (51.4–73.4)		280/341	82.9% (77.7–87.1)	84.7% (77.9–90.7)		343/364	93.9% (90.1–96.3)	96.9% (92.6–99.7)	
HLD	127/171	78.9% (71.1–85.1)	81.0% (72.4–88.7)		307/341	92.4% (88.9–94.9)	94.8% (90.2–98.5)		398/411	96.2% (91.6–98.3)	98.5% (95.8–99.9)	
Gender				0.7				0.3				0.2
Male	428/671	64.1% (60.0–68.1)	65.4% (59.3–71.3)		907/1088	83.5% (80.9–85.9)	86.0% (82.3–89.5)		1280/1351	94.5% (92.9–95.8)	97.6% (95.5–99.4)	
Female	47/71	66.8% (53.6–77.8)	68.6% (59.9–77.0)		97/113	87.5% (79.4–92.7)	90.0% (84.7–94.6)		145/149	97.3% (92.1–99.1)	99.0% (96.9–100.0)	
Grouped age				0.6				0.3				0.6
18–34	361/568	63.9% (59.0–68.5)	64.9% (58.1–71.5)		773/929	83.3% (80.2–86.0)	85.6% (81.3–89.5)		1126/1183	95.7% (94.1–96.8)	98.4% (96.3–99.9)	
35–55	114/174	66.2% (57.2–74.2)	67.2% (60.0–74.2)		231/272	86.5% (81.2–90.4)	89.1% (84.8–93.0)		299/317	94.9% (91.6–96.9)	97.6% (95.0–99.7)	
Marital status				0.9				0.4				0.2
Single	398/624	65.3% (60.2–70.0)	66.6% (60.9–72.1)		838/1003	84.1% (80.7–86.9)	86.5% (83.0–89.9)		1208/1269	95.9% (94.5–96.9)	98.8% (97.1–99.9)	
Married/Cohabiting	77/118	64.1% (51.8–74.8)	65.2% (55.1–74.8)		166/198	86.7% (81.0–90.8)	88.7% (82.8–93.7)		217/231	93.7% (88.6–96.7)	96.4% (92.4–99.4)	
Occupation				0.006				0.14				0.6
Student	152/245	61.1% (52.2–69.4)	62.4% (53.7–70.9)		300/366	85.0% (80.6–88.6)	87.6% (82.1–92.5)		504/532	94.2% (91.1–96.3)	97.9% (94.6–99.9)	
Technician/agent	136/225	58.7% (50.1–66.8)	59.3% (48.8–69.7)		348/425	79.6% (73.7–84.5)	81.5% (74.4–87.7)		411/437	95.4% (92.8–97.0)	97.2% (93.6–99.7)	
Senior	103/162	63.8% (53.3–73.2)	65.0% (54.8–74.6)		234/270	86.4% (79.3–91.4)	88.4% (82.8–93.3)		345/358	95.4% (91.0–97.7)	98.0% (95.0–99.9)	
Other	81/105	82.1% (72.5–88.8)	83.4% (73.3–92.0)		121/139	89.3% (82.3–93.7)	90.9% (83.5–96.8)		165/173	96.8% (93.1–98.6)	98.0% (94.0–99.9)	
Comorbidities				0.003				0.3				0.5
None	461/725	64.2% (59.3–68.8)	65.5% (60.4–70.4)		997/1194	84.6*	8.72% (84.1–90.0)		1413/1488	95.8*	98.3% (96.6–99.7)	
One or more	14/17	92.2% (71.5–98.2)	87.3% (64.2–99.3)		7/7	100.0*	83.0% (54.2–100.0)		12/12	100.0*	94.2% (82.6–100.0)	
Symptoms				0.14				0.087				0.9
None	319/489	67.7% (62.2–72.8)	69.0% (62.8–74.9)		816/983	83.8% (80.5–86.6)	86.2% (82.7–89.4)		1102/1158	95.4% (93.6–96.7)	98.4% (96.5–99.8)	
One or more	156/253	60.1% (51.1–68.5)	61.3% (52.9–69.5)		188/218	89.0% (83.5–92.8)	91.5% (84.9–96.7)		323/342	95.1% (91.8–97.1)	97.9% (94.5–99.9)	
Previous contact with a COVID-19 case				0.3				0.3				0.5
No/don’t know	471/733	41.0% (5.1–90.0)	66.6% (61.5–71.4)		984/1175	85.1% (82.4–87.4)	87.5% (84.5–90.4)		1409/1483	95.4% (93.8–96.5)	98.4% (96.8–99.8)	
Yes	2/6	65.2% (60.4–69.8)	50.5% (13.3–87.7)		16/21	72.0% (34.0–92.8)	75.5% (53.0–93.2)		15/16	93.7%*	85.2% (60.0–99.1)	
Previously PCR COVID-19 tested				0.2				0.2				0.3
No	415/647	65.2% (60.4–69.8)	67.5% (62.2–72.7)		874/1044	84.2% (81.1–86.9)	86.7% (83.4–89.7)		1300/1372	95.1% (93.5–96.4)	98.1% (96.4–99.6)	
Yes	60/95	58.0% (44.1–70.6)	58.8% (44.8–71.9)		130/157	88.3% (82.1–92.5)	90.6% (82.9–96.8)		125/128	97.6% (91.5–99.4)	97.9% (92.8–99.9)	
Outcome of previous test				0.4				0.4				0.7
Negative	58/94	57.6%*	58.9% (45.2–72.1)		119/144	89.5%*	89.6% (81.0–96.3)		120/123	98.3%*	96.9% (90.2–99.9)	
Positive	1/1	100.0%*	66.3% (20.7–100.0)		7/7	100.0%*	88.7% (68.1–100.0)		8/8	100.0%*	85.3% (59.6–100.0)	
COVID-19 vaccination									25/25	100.0%*	93.5% (81.2–100.0)	

*The survey confidence interval was not computed because the observed proportion was 100% or close to or no sufficient sample size

Table 4 presents the estimated AMPC by site and for the entire sample. Overall, the monthly percent change in seroprevalence was 5.58% (95% CI 4.33–6.85) in the overall population. In the CHY and JHY sites, the AMPC estimates were similar 6.55% (95% CI 5.70–7.40) and 5.95% (95% CI 4.72–7.20), respectively. However, this estimate was 3.03% (95% CI 1.44–4.66) at the LHD site.

**Table 4:** Average monthly percent change (AMPC) estimated in seroprevalence by sites and in the overall sample, Blood Donors Services, Cameroon, 2021

Site	CHY	JHY	LHD	Overall
AMPC (95% CI)	6.55% (5.70–7.40)	5.95% (4.72–7.20)	3.03% (1.44–4.66)	5.58% (4.33–6.85)

### Associated Factors of Anti-SARS-CoV-2 Antibody Seropositivity over the Periods

The weighted logistic regression was applied for each cross-sectional survey, with the same covariates, except the comorbidity covariate, which was not introduced in surveys 2 and 3, respectively. The participants in these two surveys were almost all without comorbidity conditions; thus, the weighted logistic regression estimates in such cases would not be correct.

The results of the adjusted odds ratio with their 95% confidence intervals are summarized in Table 5. In survey 1, the study site, participant occupation, and comorbidity condition were associated with anti-SARS-CoV-2 antibody seropositivity. Participants at the LHD site were at higher odds of anti-SARS-CoV-2 antibody seropositivity than those at the CHY site (adjusted OR 2.65, with 95% CI [1.57–4.49], *p* value < 0.001), while there was no difference in seropositivity odds between participants of JHY and CHY. Participants in senior position were at higher odds of anti-SARS-CoV-2 antibody seropositivity compared to students (adjusted OR 3.61, with 95% CI [1.77–7.40], *p* value < 0.001). However, there was no difference in seropositivity odds between participants in senior positions and participants in other socio-professional categories excluding students. Finally, the participants with comorbidity conditions were more likely to be seropositive compared to those without any comorbid condition (adjusted OR 6.07, with 95% CI [1.52–24.3], *p* value = 0.011).

**Table 5:** Weighted multivariate logistic regression for associated factors of seropositivity by different survey, Blood Donors Services, Cameroon, 2021

Characteristic	Survey 1				Survey 2				Survey 3			
	Adjusted OR	95% CI	*p* value	Global *p* value	Adjusted OR	95% CI	*p* value	Global *p* value	Adjusted OR	95% CI	*p* value	Global *p* value
Study site				< 0.001				< 0.001				0.3
Ref = CHY												
JHY	0.92	0.55–1.52	0.7		1.36	0.86–2.13	0.2		0.71	0.34–1.48	0.4	
LHD	2.65	1.57–4.49	< 0.001		2.96	1.82–4.83	< 0.001		1.39	0.52–3.71	0.5	
Gender				0.7				0.2				0.3
Ref = male												
Female	1.11	0.64–1.91	0.7		1.47	0.84–2.57	0.2		1.99	0.57–6.94	0.3	
Grouped age				0.5				0.9				0.7
Ref = 35–55												
18–34	1.16	0.71–1.91	0.5		0.95	0.54–1.66	0.9		1.13	0.57–2.27	0.7	
Marital status				0.6				> 0.9				0.2
Married/cohabiting												
Single	1.17	0.65–2.11	0.6		0.97	0.52–1.81	> 0.9		1.75	0.70–4.38	0.2	
Profession				0.002				0.2				0.6
Ref = student												
Technician/agent	1.21	0.73–2.03	0.5		1.40	0.82–2.38	0.2		1.14	0.58–2.26	0.7	
Senior	3.61	1.77–7.40	< 0.001		2.18	1.00–4.77	0.050		2.10	0.70–6.30	0.2	
Other	1.10	0.60–1.99	0.8		1.49	0.81–2.76	0.2		1.22	0.57–2.64	0.6	
Comorbidities				0.011								
Ref = none												
One or more	6.07	1.52–24.3	0.011									
Previous contact with a COVID-19				0.3				0.058				0.3
Ref = yes												
No/do not know	2.71	0.45–16.4	0.3		4.13	0.95–17.9	0.058		3.34	0.42–26.5	0.3	
Previously PCR COVID-19 tested				0.4				0.3				0.10
Ref = yes												
No	1.33	0.73–2.43	0.4		0.71	0.39–1.30	0.3		0.37	0.11–1.23	0.10	
Symptoms				0.3				0.10				0.6
Ref = one +												
None	1.25	0.79–1.98	0.3		0.63	0.37–1.09	0.10		1.17	0.61–2.23	0.6	
Hosmer–Lemeshow test *p* value	0.07				0.24				0.41			
Area under curve (AUC)	0.60				0.59				0.60			

In survey 2, only the study site remained associated with anti-SARS-CoV-2 antibody seropositivity, with participants at the LHD site remaining at higher odds of seropositivity than those at the CHY site (adjusted OR 2.96, with 95% CI [1.82–4.83], *p* value < 0.001), and there was still no difference in seropositivity odds between participants at JHY and CHY. Ultimately, in survey 3, there was no associated factor with anti-SARS-CoV-2 antibody seropositivity.

## Discussion

To our knowledge, this study is the first in Cameroon and one of the few studies in the literature to have determined anti-SARS-CoV-2 antibody seropositivity over three periods and two different epidemic waves. Combining our findings with those obtained from the serosurveys carried out during the first wave would provide more insight into the spread of the virus in the main urban cities of Cameroon. Such findings could be generalized to urban cities in African countries. In fact, these cities generally have similar structures in terms of urbanization process, population movement, and spatial distribution of the population [22]. Moreover, responses to COVID-19 were similar in African countries with poor adherence to non-pharmaceutical interventions and low vaccination rates.

The main relevance of our findings is both the rapid increase in anti-SARS-CoV-2 antibody seropositivity at the study sites and the change over time in the factors associated with anti-SARS-CoV-2 antibody seropositivity. The results from our third survey indicated that the spread of the virus has been generalized in the community, to a level such that no factors were associated with seropositivity. According to the results, by December 2021, almost all the adults in Yaoundé and Douala would have been infected. In Cameroon, restrictive and preventive measures were implemented as of March 18, 2020 [23], 2 weeks after the first case was detected. However, these measures lasted only a few weeks, and were relaxed on April 30, 2020 [24]. Cameroonians were found to have generally good knowledge of the COVID-19 preventive measures [25]. A study conducted from June to December 2020 indicated that adherence to these preventive measures had declined over time [26]. Although no studies evaluated this decline across our survey periods, based on the observations, adherence to preventive measures from January 2021 till date was residual among the population. This would, therefore, explain the wide spread of the virus in the population.

Considering a serosurvey conducted during the first wave in Yaoundé [8], and comparing it to our estimates for the Yaoundé sites, one would conclude that there was a rapid increase in the spread of the virus between the first and second wave of the outbreak. Ndongo et al. [9], from two consecutive serosurveys conducted in Yaoundé during January–February 2021 for the first survey and April–May 2021 for the second survey, obtained an estimated seroprevalence of 18.6% and 51.3%, respectively. These estimates were lower than our estimates obtained at similar periods at the Yaoundé sites. These differences may be due to the difference in the target population. Our surveys targeted adults, whereas theirs targeted all age groups, including children and adolescents, who are generally less susceptible to COVID-19 transmission compared to adults [27, 28].

The higher seroprevalence estimates and rapid spread of the virus in our sample may be explained in part by the place of residence of the participants. Indeed, the study sites where located in Yaoundé and Douala, which are the two main cities of Cameroon. Yaoundé and Douala are, respectively, the political and economic capitals of Cameroon, and are home to the country’s two international airports. Therefore, these two cities are the gateways for imported cases of the virus. Moreover, these cities, like many African cities, face the challenges of rapid and unplanned urbanization, which may mainly increase community spread of COVID-19 [29]. However, our results indicate that transmission was generally higher in Douala than in Yaoundé. Douala is a port city, and an industrial hub that hosts over 80% of Cameroon’s industries [30]. Thus, Douala would be more dynamic with a greater mobility of the population than Yaoundé, which could explain the greater spread of the virus in Douala compared to Yaoundé.

In our study, age was not associated with anti-SARS-CoV-2 antibody seropositivity, and this was worth noting as our sample was uniformly composed of adults. The association of age with anti-SARS-CoV-2 antibody seropositivity was due generally to children and adolescents who generally have less risk of COVID-19 infection than adults [8, 27, 28, 31, 32]. Unlike some other studies [8, 33, 34], there was no sex difference in anti-SARS-CoV-2 antibody seropositivity in our study. Most of these studies were conducted during the first wave of the outbreak when gender behavior would have particularly explained the observed sex differences in SARS-CoV-2 infection. However, our serosurveys were carried out during the second and third wave of the outbreak, when the spread of the virus became widespread in the population, regardless of the gender. These findings are consistent with other serosurveys carried out after the first wave, in which no difference was observed between men and women in the risk of SARS-CoV-2 infection [9, 12]. In our first survey, individuals with comorbidity conditions had a higher risk of seropositivity compared to those without comorbidity conditions. This finding is consistent with those obtained by Tang et al. [35]. People with comorbidity conditions would have particularly low immunity compared with those without comorbidity, and would, therefore, be more prone to SARS-CoV-2 seropositivity.

Overall, the outbreak had spread in Cameroon more than it seemed. Although mortality and severity remain low in this setting, adequate measures must be taken to prevent the continued spread of the virus. Public health authorities must develop strategies to increase the vaccination rate, which is currently very low (only 4.6% of the total population has been vaccinated [36]). Despite the current concern on breakthrough cases, which are on the rise [37, 38], the vaccine has been shown to be effective in preventing COVID-19 cases [39]. Therefore, in a setting where compliance with non-pharmaceutical intervention was not sustainable, vaccination remains the main alternative and should be implemented to prevent the continued spread of the virus within communities.

However, not taking into account the duration of natural immunity in our study could be a limitation that could affect seroprevalence levels. Indeed, antiviral antibody titers especially anti-protein *N* titers have been found to drop rapidly over time [40, 41]. The cross-sectional nature of serosurveys, even if repeated, do not allow for the detection of many seropositive cases, as discussed in Xu et al. [42]. This may explain the decrease in seroprevalence in some settings where the nucleocapsid protein antibody was targeted such as in Schoenhals et al. [43]. These issues still posing questions in the estimation of the actual size and attack rate of the outbreak. Considering a cohort or longitudinal study design in which patients will be followed-up over time with multiple testing could be important for a better estimation of the attack rate, including the re-infection rate. This could be an interesting topic for further studies.

## Conclusion

We demonstrated a generalized and homogeneous spreading of the SARS-CoV-2 virus among adults in the two capital cities of Cameroon. This suggests the ineffectiveness of the COVID-19 control measures adopted by the Cameroonian public health authorities. To avoid the long-term spreading of the virus with the development of its strains among communities, the public health authorities should implement adequate interventions, especially to increase the COVID-19 vaccination rate within the population, which is currently very low.

## Data Availability

Data are available on reasonable request. If approved, the requestor must sign a data use agreement. Additionally, the study protocol is available upon request.

## Abbreviations

AMPC: Average monthly percent change
CSSE: Center for Systems Science and Engineering
CHY: Central Hospital of Yaoundé
CI: Confidence interval
CrI: Credible interval
COVID-19: Coronavirus disease 2019
CO: Cut-off
Dos: Optical density of the sample
ELISA: Enzyme linked immunosorbent assay
Ig G: Immunoglobulin G
JHY: Jamot Hospital of Yaounde
LHD: Laquintinie Hospital of Douala
PCR: Polymerase chain reaction
SARS-CoV-2: Severe acute respiratory syndrome coronavirus 2
WHO: World Health Organization
